# Supplementary material for: Perception of physicians towards electronic prescription system and associated factors at resource limited setting 2021: Cross sectional study
Source: PLoS One. 2022 Mar 18;17(3):e0262759. doi: 10.1371/journal.pone.0262759 (PMC8932612; doi:10.1371/journal.pone.0262759)
Supplement: S1 File — (DOCX) [file pone.0262759.s001.docx]

**Questionnaire for assessment of physician’s perception towards electronic prescription**

Part 1: Socio-demographic related Questions (encircle your responses

| Item | Questions | Responses |  |
| --- | --- | --- | --- |
| 101 | Age | …………….. |  |
| 102 | Gender | 1. Female 2. Male |  |
| 103 | Educational level | 1. General practitioner 2. Resident 3. Specialist |  |
| 104 | Department | 1. Internal Medicine 2. Gynecology 3. Pediatrics 4. Surgery |  |
| 104 | Working Experience in years | -------------------- |  |

| Part 2 Current prescription status related questions | | | | |
| --- | --- | --- | --- | --- |
| Item | Question | Response | | |
|  |  | Yes | | No |
| 201 | I have legible handwriting |  |  | |
| 202 | I like paper prescription |  |  | |
| 203 | paper prescription is prone to errors |  |  | |
| 204 | I have encountered refill demand for lost prescription |  |  | |
| 205 | My prescription was altered by the patient |  |  | |
| 206 | My prescription was altered by the patient |  |  | |
| 207 | My prescription pads were stolen |  |  | |
| 208 | The pharmacist called back to clear doubts |  |  | |
| 209 | An incorrect drug was filled from the pharmacy |  |  | |
| Part 3 Current computer usage related questions | | | | |
| Item | Question | Response | | |
|  |  | Yes | | No |
| 301 | I am comfortable with computer use |  | |  |
| 302 | I use a computer for personal purpose |  | |  |
| 303 | I use a computer at home |  | |  |
| 304 | I use a computer in the hospital |  | |  |
| 305 | I have good knowledge of computer usage |  | |  |

| Part 4 technical skill related questions | | | | | | |
| --- | --- | --- | --- | --- | --- | --- |
|  | Questions | Response | | | | |
| Item | Please select the one to which your performance computer related skill is exactly matching, use the symbol in bracket to show your answer (X ) | Strongly disagree(1) | Disagree(2) | Neutral (3) | Agree  (4) | Strongly agree(5) |
| 401 | I think using a computer for an electronic prescription would make my work  more effective and accurate |  |  |  |  |  |
| 402 | I think I am good at operating computer system |  |  |  |  |  |
| 403 | I think I am fast in responding to training on new device |  |  |  |  |  |
| 404 | I think I am really motivated to pick up the new electronic prescription system |  |  |  |  |  |
| 405 | I think I have the ability to use the electronic prescription system |  |  |  |  |  |

| Part 5 Perceived usefulness related questions | | | | | | |
| --- | --- | --- | --- | --- | --- | --- |
|  | Questions | Response | | | | |
| Item | Please select the one to which your opinion is exactly matching, use the symbol in bracket to show your answer (X ) | Strongly disagree  (1) | Disagree  (2) | Neutral (3) | Agree  (4) | Strongly agree(5) |
| 501 | Using an electronic prescription system will decrease the cost of healthcare |  |  |  |  |  |
| 502 | Using an electronic prescription system promote the use of data for research |  |  |  |  |  |
| 503 | The electronic prescription system will provide an alert when the patient  receives medication |  |  |  |  |  |
| 504 | The electronic prescription system will improve alert about the drug |  |  |  |  |  |
| 505 | The electronic prescription system will save time and reduce error |  |  |  |  |  |
| 506 | The electronic prescription system will be safe |  |  |  |  |  |

| Part 6 Organizational related questions | | | | | | |
| --- | --- | --- | --- | --- | --- | --- |
|  | Questions | Response | | | | |
| Item | Please select the one to which your opinion about your organization exactly matching, use the symbol in bracket to show your answer (X ) | Strongly disagree  (1) | Disagree  (2) | Neutral (3) | Agree  (4) | Strongly agree(5) |
| 601 | I think the hospital firm can afford to improve the internet network |  |  |  |  |  |
| 602 | I think the internet network in the hospital is good |  |  |  |  |  |
| 603 | I think the hospital firm can afford to get all physicians computer |  |  |  |  |  |
| 604 | The hospital firm Can manage and be consistent with the system over a long  time |  |  |  |  |  |
| 605 | I think the hospital firm would be ready to adopt the new system based on the  infrastructural facilities |  |  |  |  |  |
| 606 | I think the patient will be ready to adopt the new system |  |  |  |  |  |
