# Supplementary material for: Perception of physicians towards electronic prescription system and associated factors at resource limited setting 2021: Cross sectional study
Source: PLoS One. 2022 Mar 18;17(3):e0262759. doi: 10.1371/journal.pone.0262759 (PMC8932612; doi:10.1371/journal.pone.0262759)
Supplement: Attachment — Submitted filename: Reviewer Comment_PONE-D-21-14066.pdf [file pone.0262759.s003.pdf]

PONE-D-21-14066
Perception of physicians towards electronic prescription system and associated factors at resource limited setting 2021: Cross sectional study

Reviewer's Comment

General Comments:
Throughout the manuscript, there are numerous grammatical faults. It is strongly recommended that the work be edited by a native English speaker or a company that provides editing services.

Key words: The authors must ensure that the keywords are MeSH words and that they are in alphabetical order.

Introduction:
According to the findings of this study, 89 percent of participants believe that paper-based prescriptions are prone to errors, however the literature mentioned [6] reveals that paper-based prescriptions have been a favored communication method for many years for physicians.

Isn't it true that if a paper-based prescription is prone to drug errors, it shouldn't be preferred?

Several references have been provided discussing paper-based versus electronic prescriptions, but despite several studies conducted on the topic in various regions of the world, there is not a single source about physicians' perceptions of electronic prescriptions.

Methods:
Incorporating a formula for calculating sample size would be more useful.

A total of 17 referral hospitals were chosen, with each receiving a proportional allotment. What was the foundation for the proportional allocation and how was it done?

"Do you have legible handwriting?" was asked as a yes or no question to assess current prescription practice. I believe that observing written prescriptions rather than asking questions is a better way to evaluate this assertion.

Technical skill was assessed using questions like: do you think using a computer for an electronic prescription would make your work more effective and accurate?, are you good at operating a computer system?, Are you fast in responding to training on a new device? Are you really motivated to pick up the new electronic prescription system (1. Strongly disagree, 2. Disagree 3. Neutral 4. Agree 5. . Strongly agree). How might these types of questions be used to measure technical skills? For determining attitude or perception, likert scale questions are utilized.

More information on the expert panel (10 doctors), such as their qualifications, departments, and so on, should be included.

What necessary correction were done in questionnaire based on the pre-test finding?
Who gave two-days training for five data collectors) on the objective of the study and data collection procedures? Who were the data collectors? How were their levels of education, experience, and other factors matched to ensure that the data they collected was uniform?

Please specify the number and date of the ethical approval.

Results:
Table 5 might be presented in a more appealing manner.

Discussion:
In light of the outcomes of this study, this area demands greater critique and clarity.

Conclusions:
Availability of data and materials: It is recommended that they be made available in the journal's repository.

References:
Check the journal's requirements for references and their citations in the article.
