# Supplementary material for: Perception of physicians towards electronic prescription system and associated factors at resource limited setting 2021: Cross sectional study
Source: PLoS One. 2022 Mar 18;17(3):e0262759. doi: 10.1371/journal.pone.0262759 (PMC8932612; doi:10.1371/journal.pone.0262759)
Supplement: Attachment — Submitted filename: PONE-D-21-14066. after review.pdf [file pone.0262759.s005.pdf]

PLOS ONE

Perception of physicians towards electronic prescription system and associated factors at resource limited setting 2021: Cross sectional study

--Manuscript Draft--

**Manuscript Number:**	PONE-D-21-14066
**Article Type:**	Research Article
**Full Title:**	Perception of physicians towards electronic prescription system and associated factors at resource limited setting 2021: Cross sectional study
**Short Title:**	Perception of physicians towards electronic prescription system and associated factors
**Corresponding Author:**	Gizaw Hailiye, MPH<br>Debre Markos University College of Health Science<br>Debre Markos, Amhara ETHIOPIA
**Keywords:**	
**Abstract:**	The health care industry has become increasingly concerned about patient safety, one of the major threats to patient safety is medical error which is among the leading cause of death globally. This medical error is even more serious in developing countries that lack a technologically supported healthcare system. In Ethiopia because of the traditional paper-based prescription system the overall medication prescribing error rate of 58.07%, in which incomplete prescriptions and dosing errors were the most prevalent error types which could be prevented if there were an electronic prescription system. Thus is study was conducted to assess physician's perception towards electronic prescription system implementation. Methods: An institution-based cross-sectional study design was conducted among physicians working at public hospitals in the Amhara region, Ethiopia, from February 1 to April 5, 2021. A simple random sampling method was used to select 384 physicians. A self-administered questionnaire was used to collect the data and analyzed using SPSS, version 21. Binary and multivariable logistic regression analysis was performed to assess factors associated with a positive perception among physicians. A value of P<.05, corresponding to a 95% CI, was considered statistically significant. The validity of the questionnaire was determined based on the view of experts and the reliability of it obtained by calculating the value of Cronbach alpha (α=.78) Results: In this study (231, 76.5% %) of the study participant had a positive perception towards electronic prescription. Around 70.8% had computer usage experience for more than 5 years, 23.7% for 1-5 years, and the rest, less than one year. About 90% of the participant claimed that their prescriptions to be legible, on the other hand, 89% perceive that paper-based prescription is prone to error. According to multivariable logistic regression analysis, Technical skill (adjusted odds ratio [AOR] 4.7, 95% CI (1.27-17.41), good internet access (AOR 2.82, 95% CI 1.75-4.54), and perceived usefulness e-prescription system (AOR 3.31, 95% CI 1.01-12.12) were significantly associated with perception. Conclusions: Most of the respondents have a positive perception of electronic prescription. Organizational factors, internet access, perceived usefulness of the system, and technical skill are the most notable factors that are associated with the perception of physicians.
**Order of Authors:**	Gizaw Hailiye, MPH
**Additional Information:**	
**Question**	**Response**
**Financial Disclosure**<br><br>Enter a financial disclosure statement that describes the sources of funding for the work included in this submission. Review the submission guidelines for detailed requirements. View published research articles from *PLOS ONE* for specific	The author(s) received no specific funding for this work.

Powered by Editorial Manager® and ProduXion Manager® from Aries Systems Corporation

examples.<br><br>This statement is required for submission and **will appear in the published article** if the submission is accepted. Please make sure it is accurate.<br><br>**Unfunded studies**<br>Enter: *The author(s) received no specific funding for this work.*<br><br>**Funded studies**<br>Enter a statement with the following details:<br>• Initials of the authors who received each award<br>• Grant numbers awarded to each author<br>• The full name of each funder<br>• URL of each funder website<br>• Did the sponsors or funders play any role in the study design, data collection and analysis, decision to publish, or preparation of the manuscript?<br>• **NO** - Include this sentence at the end of your statement: *The funders had no role in study design, data collection and analysis, decision to publish, or preparation of the manuscript.*<br>• **YES** - Specify the role(s) played.<br><br>* typeset	
**Competing Interests**<br><br>Use the instructions below to enter a competing interest statement for this submission. On behalf of all authors, disclose any competing interests that could be perceived to bias this work—acknowledging all financial support and any other relevant financial or non-financial competing interests.<br><br>This statement is required for submission and **will appear in the published article** if the submission is accepted. Please make sure it is accurate and that any funding sources listed in your Funding Information later in the submission form are also declared in your Financial Disclosure statement.	The authors have declared that no competing interests exist.

View published research articles from [PLOS ONE](#) for specific examples.<br><br>**NO authors have competing interests**<br><br>Enter: *The authors have declared that no competing interests exist.*<br><br>**Authors with competing interests**<br><br>Enter competing interest details beginning with this statement:<br><br>*I have read the journal's policy and the authors of this manuscript have the following competing interests: [insert competing interests here]*<br><br>* typeset	
**Ethics Statement**<br><br>Enter an ethics statement for this submission. This statement is required if the study involved:<br><br>• Human participants<br>• Human specimens or tissue<br>• Vertebrate animals or cephalopods<br>• Vertebrate embryos or tissues<br>• Field research<br><br>Write "N/A" if the submission does not require an ethics statement.<br><br>General guidance is provided below. Consult the submission guidelines for detailed instructions. **Make sure that all information entered here is included in the Methods section of the manuscript.**	NA

**Format for specific study types**<br><br>**Human Subject Research (involving human participants and/or tissue)**<br>• Give the name of the institutional review board or ethics committee that approved the study<br>• Include the approval number and/or a statement indicating approval of this research<br>• Indicate the form of consent obtained (written/oral) or the reason that consent was not obtained (e.g. the data were analyzed anonymously)<br><br>**Animal Research (involving vertebrate animals, embryos or tissues)**<br>• Provide the name of the Institutional Animal Care and Use Committee (IACUC) or other relevant ethics board that reviewed the study protocol, and indicate whether they approved this research or granted a formal waiver of ethical approval<br>• Include an approval number if one was obtained<br>• If the study involved *non-human primates*, add *additional details* about animal welfare and steps taken to ameliorate suffering<br>• If anesthesia, euthanasia, or any kind of animal sacrifice is part of the study, include briefly which substances and/or methods were applied<br><br>**Field Research**<br><br>Include the following details if this study involves the collection of plant, animal, or other materials from a natural setting:<br>• Field permit number<br>• Name of the institution or relevant body that granted permission	
**Data Availability**<br><br>Authors are required to make all data underlying the findings described fully available, without restriction, and from the time of publication. PLOS allows rare exceptions to address legal and ethical concerns. See the PLOS Data Policy and FAQ for detailed information.	Yes - all data are fully available without restriction

A Data Availability Statement describing where the data can be found is required at submission. Your answers to this question constitute the Data Availability Statement and **will be published in the article**, if accepted.<br><br>**Important:** Stating 'data available on request from the author' is not sufficient. If your data are only available upon request, select 'No' for the first question and explain your exceptional situation in the text box.<br><br>Do the authors confirm that all data underlying the findings described in their manuscript are fully available without restriction?	
**Describe where the data may be found in full sentences. If you are copying our sample text, replace any instances of XXX with the appropriate details.**<br><br>• If the data are **held or will be held in a public repository**, include URLs, accession numbers or DOIs. If this information will only be available after acceptance, indicate this by ticking the box below. For example: *All XXX files are available from the XXX database (accession number(s) XXX, XXX.*).<br>• If the data are all contained **within the manuscript and/or Supporting Information files**, enter the following: *All relevant data are within the manuscript and its Supporting Information files.*<br>• If neither of these applies but you are able to provide **details of access elsewhere**, with or without limitations, please do so. For example:<br><br>*Data cannot be shared publicly because of [XXX]. Data are available from the XXX Institutional Data Access / Ethics Committee (contact via XXX) for researchers who meet the criteria for access to confidential data.*<br><br>*The data underlying the results presented in the study are available from (include the name of the third party*	All relevant data are within the manuscript and its Supporting Information files.

and contact information or URL).<br>• This text is appropriate if the data are owned by a third party and authors do not have permission to share the data.<br><br>* typeset	
Additional data availability information:	Tick here if the URLs/accession numbers/DOIs will be available only after acceptance of the manuscript for publication so that we can ensure their inclusion before publication.

Powered by Editorial Manager® and ProduXion Manager® from Aries Systems Corporation

Click here to access/download;Manuscript;Manuscript.pdf

Original Paper

Perception of physicians towards electronic prescription system and associated factors at resource limited setting 2021: Cross sectional study

Gizaw Hailiye[1*] Maru Meseret[1], Bayou Tilahun[1], Zegeye Regasa[1], Tewodros Eshetie[1], Mohammedjud Hassen[2], Samuel Hailegebriel[3]

[1]Department of health informatics College amedicine and health science Debre Markos University, Debre Markos Ethiopia

[2]Department of health informatics College of Health science Mettu University, Mettu Ethiopia.

[3]Department of Health Informatics College of Medicine and Health science Arba Minch University, Arba Minch Ethiopia.

Corresponding Author: Gizaw Hailiye

Telephone: +251921493560

Email Address: ghailiye463@gmail.com

Abstract

The health care industry has become increasingly concerned about patient safety, one of the major threats to patient safety is medical error which is among the leading cause of death

globally. This medical error is even more serious in developing countries that lack a technologically supported healthcare system. In Ethiopia because of the traditional paper-based prescription system the overall medication prescribing error rate of 58.07%, in which incomplete prescriptions and dosing errors were the most prevalent error types which could be prevented if there were an electronic prescription system. Thus is study was conducted to assess physician's perception towards electronic prescription system implementation.

Methods: An institution-based cross-sectional study design was conducted among physicians working at public hospitals in the Amhara region, Ethiopia, from February 1 to April 5, 2021. A simple random sampling method was used to select 384 physicians. A self-administered questionnaire was used to collect the data and analyzed using SPSS, version 21. Binary and multivariable logistic regression analysis was performed to assess factors associated with a positive perception among physicians. A value of P<.05, corresponding to a 95% CI, was considered statistically significant. The validity of the questionnaire was determined based on the view of experts and the reliability of it obtained by calculating the value of Cronbach alpha (α=.78)

Results: In this study (231, 76.5% %) of the study participant had a positive perception towards electronic prescription. Around 70.8% had computer usage experience for more than 5 years, 23.7% for 1-5 years, and the rest, less than one year. About 90% of the participant claimed that their prescriptions to be legible, on the other hand, 89% perceive that paper-based prescription is prone to error. According to multivariable logistic regression analysis, Technical skill (adjusted odds ratio [AOR] 4.7, 95% CI (1.27-17.41), good internet access (AOR 2.82, 95% CI 1.75-4.54), and perceived usefulness e-prescription system (AOR 3.31, 95% CI 1.01-12.12) were significantly associated with perception.

Conclusions: Most of the respondents have a positive perception of electronic prescription. Organizational factors, internet access, perceived usefulness of the system, and technical skill are the most notable factors that are associated with the perception of physicians.

Keywords electronic prescription, system, physician, perception.

Introduction

The health care industry has become increasingly concerned about patient safety, which corresponds to societal trends [1]. Medication safety is one of the most important concerning issue in global health policy[1] . Medical error is a common encounter and represents an important public health problem posing a serious threat to patient safety. A study released in 2016 found that medical error is the third leading cause of death in the United States, after heart disease and cancer. Researchers looked at studies that analysed the medical death rate data from 2000 to 2008 and extrapolated that over 250,000 deaths per year had stemmed from a medical error, which translates to 9.5% of all deaths annually in the United States of America[2].

Medication errors are the leading causes of avoidable patient harm in the health care system across the world. In African health care, setting medication errors are common health problems [3].

According to the systematic review and meta-analysis study conducted in Ethiopia, the overall medication error in Ethiopia was found to be 57.6%, while the magnitude of medication administration and prescription error was 58.4%[4].

A study done in the northeast part of the country (Dessie referral hospital) indicated that the overall medication prescribing error rate of 58.07%, in which incomplete prescriptions and dosing errors were the most prevalent error types which could be prevented if there were electronic prescription system[5]. Paper-based prescription has been a preferred communication method for physicians in decisions relating to medication therapy and for pharmacists to dispense medications. It is also considered as a valuable resource for the patients on how to use the medicine to achieve the maximum benefit[6]. However, literature revealed that electronic prescription is more reliable when it comes to patient safety than the traditional paper-based prescription method[7].

The use of e-prescriptions has the potential to improve the quality of patient care at the pharmacy[1]. Indeed, studies show significant improvements associated with e-prescription implementation, including an 86% decrease in serious medication errors, and an increase in Medicare formulary adherence from 14% to 88% [8]. Electronic prescription is an alternative to many years old paper-based prescription. Electronic prescribing and dispensing processes of drugs whether in medical practice, follow up or research has become an integral part of pharmacoinformatics[9].

Even though the electronic prescription system is an essential tool for the healthcare sector, the adoption and utilization of electronic prescription remain low in developing countries[10]. A lot of healthcare organizations across the globe have implemented electronic information systems to improve the information recording process, but only a few have been successful[11].

The percentages of failures are alarmingly higher in adoption to meet the desired benefits from the implementation. Globally, more than 50% of electronic information systems including electronic prescription projects failed before they reached their target[12]. Numerous reasons are given for low electronic system adoption such as resistance, the opposition of transition from paper-based to the electronic system, technical competency of the frontline users, and ease of use

of a given electronic system, lack of organizational readiness, and security and confidentiality issue[12][13][14][15][16].

One of the major contributing factors in the successful implementation of electronic prescription is health care professional's technical competency[14][17]. In addition to the physician competency to use e-prescription error, free e-prescription requires regular technical assistance[14][16][18].

The infrastructure of a given health care organization and the culture of the society in which the organization operates govern the readiness of the professional to implement e-prescription[19]. In addition to this, the overall readiness of healthcare organizations to implement e-prescription is affected by individual-related factors[20] . According to the Technology Acceptance model ease of use of a given information system influences users' perception and outcomes[14][15].

The other factor that governs the perception of the physician to use electronic prescription systems is the security concern of electronic systems. Electronically transmitted data could be attacked and be used for other criminal purposes such as inappropriate prescribing of controlled substances or high-cost medications[21]. While most e-prescribing systems do have safeguards in place to protect access, they are susceptible to attack.

As successful adoption of electronic prescription system or any electronic health information system is a change process it needs many behavioural modifications in the work environment for physicians[22].

Perception assessment, as a comprehensive measure to provide a proper image of existing conditions and the preparedness of healthcare organizations to change, is also a way to identify potential causes of failure in innovation[23]. This study, therefore, aims to assess the perception of physicians towards electronic prescription system implementation and associated factors at public hospitals in East and West Gojjam zones North Ethiopia.

Method

Study design and setting

This was a cross-sectional, questionnaire-based study done to assess physician's perception towards implementation of an electronic prescription system and associated factors at public health hospitals in East and West Gojjam zones north Ethiopia 300km from Addis Ababa

The sample size was computed as 384 which was 80% of the total populatio[illegible]uring the data collection period, using single population proportion formula taking 50% at 95% confidence

level assuming a 5% margin of error and 10% for non-response rate. There are 17 hospitals in the west and east Gojjam zones and all the 17 referral hospitals were taken and then proportional allocation was made for each hospital finally simple random sampling method was used. 384 participants were recruited using the lottery method (each member of the population was assigned a unique number and the number was written on separate paper/card/ with the same size, the card mixed in the basket well and sample drown) and formed the sample.

The survey consisted of 35 questions encompassing the following domains: 1. socio-demographic characteristics, 2. Current prescription status, 3. Current computer usage status 4.physician technical skill, 5. Perceived usefulness, and 7. Organizational factors. The current prescription practice was assessed using yes or no questions as follows: Do you have legible handwriting, Do you like paper prescription, a paper prescription is prone to errors, Do you have encountered refill demand for lost prescription (Yes, No) Current computer usage status was assessed as follows: Are you comfortable with computer use? , do you use a computer for personal purposes? , Do you use a computer at home?, do you use a computer in the hospital (Yes, No)

Technical skill was assessed using questions like: do you think using a computer for an electronic prescription would make your work more effective and accurate?, are you good at operating a computer system?, Are you fast in responding to training on a new device? Are you really motivated to pick up the new electronic prescription system (1. Strongly disagree, 2. Disagree 3. Neutral 4. Agree 5. . Strongly agree)

Perceived usefulness of electronic prescription was assessed as follows, Using an electronic prescription system will decrease the cost of healthcare, Using an electronic prescription system promote the use of data for research, The electronic prescription system will provide an alert when the patient receives medication, The electronic prescription system will improve alert about the drug, The electronic prescription system will save time and reduce error, The electronic prescription system will be safe, (1. Strongly disagree, 2. Disagree 3. Neutral 4. Agree 5. . Strongly agree). Organizational related factors were assessed using questions like: do you think the hospital firm can afford to improve the internet network, do you think the internet network in the hospital is good, do you think the hospital firm can afford to get all physicians computer, The hospital firm Can manage and be consistent with the system over a long time, do you think the hospital firm would be ready to adopt the new system based on the infrastructural facilities, do

you think the patient will be ready to adopt the new system(1. Strongly disagree, 2. Disagree 3. Neutral 4. Agree 5. . Strongly agree)

A self-administered questionnaire was adapted from the previous studies [26],[15]. To ensure the validity of the questionnaire, an expert panel (10 doctors having at least 5 years' experience in general practice or primary care research) was invited to review the tool and revise it and reliability was calculated to be α = 0.78.

Before the actual data collection, pilot testing of the questionnaire was conducted among 20 physicians at Debre tabor hospital to check internal consistency within the questioners. Then necessary correction was done based on the pre-test finding.

Two-days training was given for (five data collectors) on the objective of the study and data collection procedures. Data were collected from February 5 to March 30, 2021, using a self-administered questionnaire, one data collector was assigned for each hospital and the supervisor facilitated the data collection process. The principal investigator and supervisors did daily supportive supervision on data collectors. Data backup activities, like storing data at different places and putting data in different formats (hard and soft copies) were performed to prevent data loss.

Study Variables and Operational Definitions

Dependent Variable

The dependent variable was perception of physician's towards electronic medical prescription system.

Independent Variables

The independent variables were sociodemographic factors (age, sex, profession, educational status, experience), system related factors (perceived usefulness, perceived ease of use), organizational factors (infrastructure), and behavioral factors (knowledge, technical skill, IT-related experience).

Operational Definitions

In this study, "physician" includes general practitioners, residents, dentists, specialists, and subspecialists.

Data Processing and Analysis

Data was entered using Epi-info version 7 and analysed using Statistical Package for Social Science (SPSS) version 20. Descriptive analyses were computed for the dependent variable in the study to determine the Perceptions of Physician to implement electronic prescription (e-prescription). Adjusted odds ratios were used to measure the association of dependent and independent variables, 95% confidence intervals, and P-value was calculated to evaluate statistical significance. Binary and multivariable logistic regression analyses were carried out to assess the effect of selected variables on perception to implement e-prescription. Standardized coefficient and 95% confidence intervals were calculated for each of the independent variables in simple binary regression models with the perception of physicians to use electronic prescription as the dependent variable.

Ethical consideration

In conducting the study, ethical clearance was obtained from the Debre Markos university ethical review board. Additional permissions to access participants were also obtained from each hospital administrator. In addition, written informed consent was gained from all participants, participation in the study was voluntary, and no incentive was provided for the participants.

Result

Socio-demographic characteristics of participants

A total of 302 physicians were included in this study with a response rate of 78.6%. About one third 225(74.5%) of the respondents were male. The mean age was 28+_3.6SD years with the majority of the age group were 25-34. About 231(76.5%) of the physicians had positive perception towards electronic prescription system.

Table 1: Sociodemographic characteristics of Participants (n= 302)

VARIABLE	CATEGORY	FREQUENCY (#)	PERCENTAGE (%)
GENDER	Male	225	74.5
	Female	77	25.5
AGE	<=30	165	54.7
	>30	137	45.3
EDUCATIONAL STATUS	General practitioner	240	79.5
	Resident	39	12.9
	Specialist	23	7.6
DEPARTMENT	Internal medicine	105	34.7
	Pediatrics	54	17.9
	Surgery	59	19.5
	Gynecologist	56	18.5
	Dermatology	14	4.6

	Other	14	4.6
WORK EXPERIENCE	1-3 years	192	63.6
	3-6 years	87	28.8
	>6 years	23	7.6

Around 70.8% had computer usage experience for more than 5 years, 23.7% for 1-5 years and the rest less than one year. As shown in Table 2, 97.3% were comfortable with use of computers, and only 52% had used computers at hospital. Of the 70.5% (181) who have heard of e-prescriptions only 14% (37) had hands on exposure in generating it.

Table 2 Response of physician on current computer usage status (N=302)

Status	Responses (%)	
	Yes	No
I am comfortable with computer use	293 (97.3%)	9(2.7)
I use computer for personal purpose	297 (98.7%)	5(1.3)
I use computer at home	276 (91.3%)	26(8.7)
I use computer at hospital	155 (51.3%)	147(48.7)
I have good knowledge of computer usage	295 (97.7%)	7(2.3)

The current prescribing activities of the physicians are summarized in Table [illegible]bout 90% the participa[illegible]aimed that their prescriptions to be legible and 76% liked paper prescriptions, on the other hand 89% perceive that paper based prescription is prone to error Table 2.

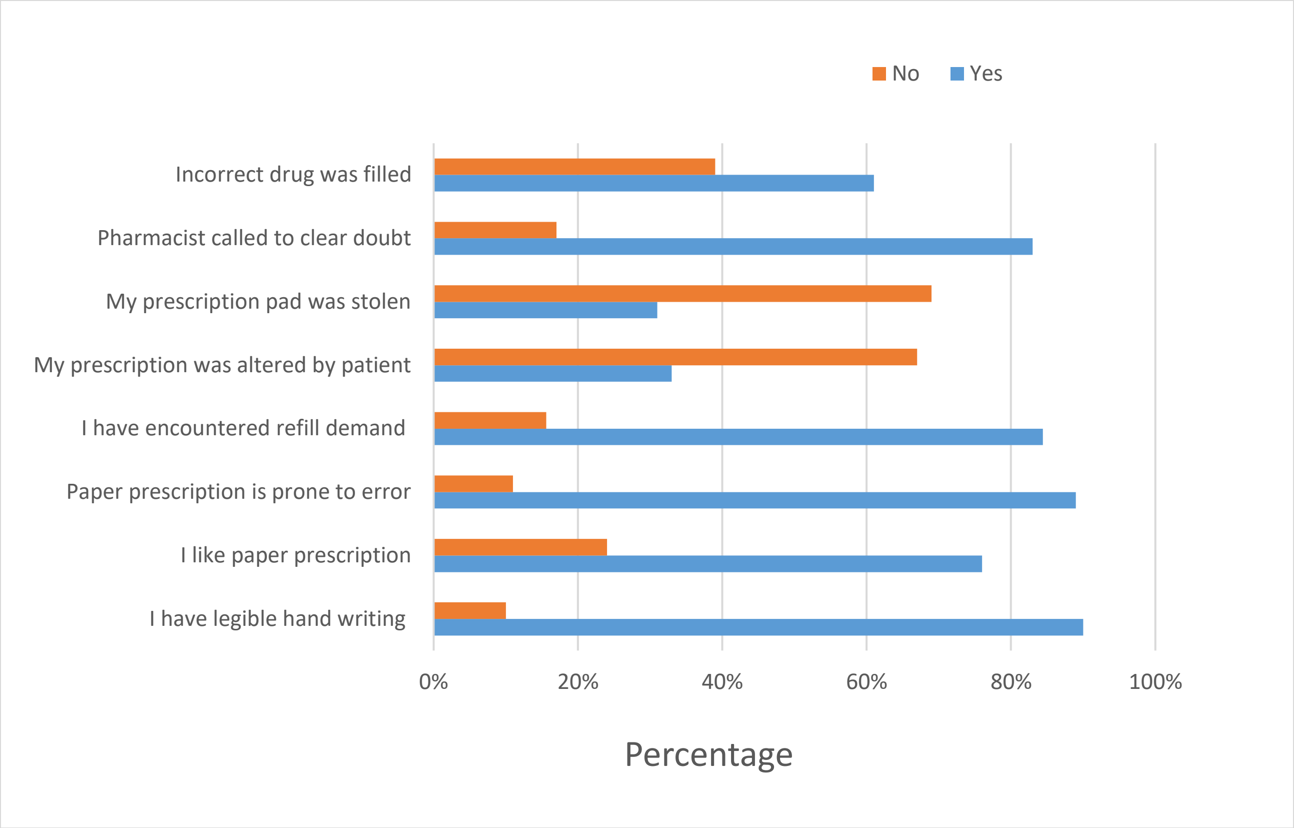

Figure 1 current prescription profile of participants (N=301).

The mean perceived usefulness and technical skill of physicians were 4±0.4, 3.9±0.45, respectively. As shown in table 3 below, majority of the participants perceive that using e-prescription system will decrease cost of healthcare (71.5%) and would promote use of data for research(91%%)

Table 3 Perception of physicians towards use of electronic prescription system (N=302)

Variable	Disagree(1+2)	Neutral(3)	Agree (4+5)
Using an electronic prescription system will decrease the cost of healthcare	7.6%	20.6%	71.5
Using an electronic prescription system promote the use of data for research	2%	7%	91%
The electronic prescription system will provide an alert when the patient receives medication	3%	16%	80%
The electronic prescription system will improve alert about the drug	0	6.3%	94.7
The electronic prescription system will save time and reduce error	3%	18%	79%

The electronic prescription system will be safe	3.3%	18.3%	78.4%

Table 4 depicts the perceived readiness of institution to implement e-prescription. Even though majority of the participants agreed to accept e-prescription once it was adopted in the institution, 40% felt that the hospital firm cannot afford to get all physicians computer. There was a compelling demand for infrastructure development and massive training in the Institution. Only 34% of the participants felt that the patient will be ready to adopt the e-prescription system.
Table 4

Table 4 perceived readiness of organization to implement e-prescription (N=302)

Variable	Disagree(1+2)	Neutral(3)	Agree(4+5)
I think the hospital firm can afford to improve the internet network	19.6%	29.6%	50.8%
I think the internet network in the hospital is good	45.5%	16.3%	38.2
I think the hospital firm can afford to get all physicians computer	40%	36.2%	23.9%
The hospital firm Can manage and be consistent with the system over a long time	22.3%	28.2%	49.5%
I think the hospital firm would be ready to adopt the new system based on the infrastructural facilities	25%	45%	30%
I think the patient will be ready to adopt the new system	14%	42%	34%

Factors Associated With perceptions Physicians towards electronic prescription

A total of 4 variables were selected as potential predictors for perception after bivariable logistic regression and entered multivariable logistic regression. Included variables were Technical skill of physicians, internet access the organizatio[illegible]ender, work experience and, perceived usefulness of the electronic prescription. Technical skill of the physicians, and availability[illegible]ernet access, perceived usefulness of electronic prescription, and organizational factors were variables which were positively related to perception of physicians towards electronic prescription

The multivariable analysis of logistic regression pointed out Technical, internet access, and perceived usefulness of e-prescription system were significantly associated with Perception.

Table 5 Bivariable and multivariable logistic regression analysis of factors with perception among physicians at public health hospitals in Amhara region Northwest Ethiopia 2021(N=302)

**Variable**		**Perception, n (%)**		**Crude OR[a] (95% CI)**	***P* value**	**AOR[b] (95% CI)**	***P* value**
		Positive	Negative				
Current computer usage							
	Yes	269 (89)	19 (.062)	3.86 (1-15.02)	.052	2.06 (0.51-8.31)	
	No	11 (0.04)	3 (.01)	1		1	
Internet access							
	Yes	162 (54)	2 (.66)	13.846 (3.174-2.25)	.000	2.82 (1.75-4.50)	**<.001**
	No	117 (38.7)	20 (6.6)	1		1	
Technical skill							
	Yes	268 (88.7)	12 (3.9)	8.75 (2.8-25.21)		4.7 (1.27-17.41)	**.02**
	No	16(5.3)	6 (2)	1		1	
Perceived usefulness							
	Yes	265 (87.7)	16(5.3)	6.62 (2.3-19.3)		3.31 (1.01-12.12)	**.041**
	No	15 (5)	6 (2)	1		1	

Organizational factor							
	Yes	157(52)	7(0.23)	2.75(1.09-6.9)	.032	1.8(.66-5.07)	
	No	122(40.4)	15(5)	1	1		

[a]OR: odds ratio.

[b]AOR: adjusted odds ratio.

Discussion

This study was conducted to assess physician’s perception towards electronic prescription system implementation in the context of Resource-limited setting hospitals. Insights were provided into future successful implementation of electronic prescription system since perception of the user greatly affect it. The study was done amongst the prescribing doctors at public health hospitals which has a high workload with physicians to population ratio of .0769/1000[24]. The study revealed that almost all 89% perceive that paper-based prescription is prone to error and 84% of the respondents encountered refill demand due to the absence of data verification and validation in paper-based prescription.

The mean perceived usefulness and technical skill of physicians were 4±0.4, 3.9±0.45, respectively this result is a little bit higher than the previous study conducted in India which was 3.5 and 3.3[25]. This might be due to the time gap between the current study and the previous study since the

previous study was conducted five years ago (20o16). Factors like internet access, perceived usefulness, the technical skill of physicians, and past computer-related training were found to be associated with the perception of physicians towards e-prescription. In this study, more than two-third 76.5% (95% CI 71.5-81) of the study participants had a positive perception towards e-prescription. This result is consistent with that of a study done in Jordan (74.7%)[26].

Different studies conducted on technology acceptance in different domains have suggested that Perceived usefulness as the main determinant factor for new technology acceptance and utilization[27][28][29]. The finding of the current study was consistent with this studies. The current study revealed that the perceived usefulness of the e-prescription system is positively associated with the positive perception of physicians 3.31(95% CI 1.01-12.12). The odds of physicians who thought the e-prescription system as useful to have positive perception is 3.31 times more likely than their counterparts. The possible reason for this could be as the user thought the system would be useful more likely to have a positive perception.

According to this study, physicians who were working in an institution with internet access (WiFi) were 2.82 times more likely to develop positive perceptions than those who had no internet access (AOR 2.82, 95% CI 1.75-4.50).

The majority of authors who conducted a study on electronic information systems acknowledge that users' perception more likely to be affected by their computer skills [14][25][29][30]. The result of this study is consistent with those findings, this study revealed that physicians who had good technical skills were 4.7 times more likely to have a positive perception towards implementation of e-prescription than those who had poor technical skills (AOR 4.7, 95% CI 1.27-17.41]).

This study summarizes the perceptions of front-line prescribers (physicians) as a pre-implementation assessment. Even though the study population corresponds to a usual proportion of prescribers in the institution a larger sample size would have yielded better results.

Conclusion

The findings of this study showed that positive perception of physicians towards electronic was 76.5%. Good internet access, perceived usefulness of e-prescription system, and the technical skill of the physicians were the most notable factors that were associated with positive perception.

According to this study finding, perception of the user has significant impact on successful

implementation of e-prescription system. To be successful in e-prescription system implementation, health care organizations should improve computer technical skill of physicians providing training on digital device usage, and improve internet connectivity.

Abbreviation and Acronyms

AOR	adjusted odds ratio
CI	Confidence interval
e-prescription	Electronic prescription
M-health	Mobile Health.
SPSS	Statistical Package for Social Science

Acknowledgment

We would like to thank Debre Markos University institute of public health for the approval of the ethical clearance, Respective hospitals and directors for giving us permission to collect data. We also forward gratitude to data collectors, supervisors and study participants.

Funding

No funding was received

Availability of data and materials

Data will be available upon request from the corresponding author

Authors' contributions

GH, conceived of the study, coordinate data collection. GH, BT, TE, MH, ZR, MM, SH performed statistical analysis and drafted the manuscript. All authors read and approved the final manuscript.

Consent for publication

Not applicable.

Disclosure

No competing interests.

Reference

1. Kauppinen H, Ahonen R, Timonen J. The impact of electronic prescriptions on medication safety in Finnish community pharmacies: A survey of pharmacists. Int J Med Inform [Internet]. 2017;100:56–62. Available from: http://dx.doi.org/10.1016/j.ijmedinf.2017.01.014

2. Makary MA, Daniel M. Medical error—the third leading cause of death in the US. Bmj. 2016;353.

3. Yousif MA, Nizar S, Elmustafa MO, Mustafa MI, Bella MM. Investigation of medication prescribing errors in Wad Medani, Gezira, Sudan. Int J Risk Saf Med. 2011;23:11–6.

4. Endalamaw A, Dessie G, Netsere H, Belachew A, Amare D, Workineh Y, et al. Medication Errors in Ethiopia: Systematic Review and Meta-Analysis. 2020.

5. Zeleke A, Chanie T, Woldie M. Medication prescribing errors and associated factors at the pediatric wards of Dessie Referral Hospital, Northeast Ethiopia. Int Arch Med [Internet]. 2014;7(1):18. Available from: https://doi.org/10.1186/1755-7682-7-18

6. Hellström L, Waern K, Montelius E, Åstrand B, Rydberg T, Petersson G. Physicians' attitudes towards ePrescribing–evaluation of a Swedish full-scale implementation. BMC Med Inform Decis Mak. 2009;9(1):1–10.

7. Pizzi LT, Suh D-C, Barone J, Nash DB. Factors related to physicians' adoption of

electronic prescribing: results from a national survey. Am J Med Qual. 2005;20(1):22–32.

8. Porterfield A, Engelbert K, Coustasse A. Electronic prescribing: improving the efficiency and accuracy of prescribing in the ambulatory care setting. Perspect Heal Inf Manag [Internet]. 2014 Apr 1;11(Spring):1g-1g. Available from: https://pubmed.ncbi.nlm.nih.gov/24808808

9. Neoh CF, Zainal INA, Hameed MA, Khan TM, Ming LC. Development and progress of pharmacoinformatics in pharmaceutical and health sciences. J Young Pharm. 2015;7(3):155.

10. eHealth WHOGO for. Atlas of EHealth Country Profiles: The Use of EHealth in Support of Universal Health Coverage: Based on the Findings of the Third Global Survey on EHealth, 2015. World Health Organization; 2016.

11. HASANAIN RA, COOPER H. Solutions to overcome technical and social barriers to electronic health records implementation in Saudi public and private hospitals. J Health Inform Dev Ctries. 2014;8(1).

12. Ketikidis P, Dimitrovski T, Lazuras L, Bath PA. Acceptance of health information technology in health professionals: an application of the revised technology acceptance model. Health Informatics J. 2012;18(2):124–34.

13. Omotosho A, Adegbola O, Adebo A. A patient-based hospital referral decision support system. Int J Comput Appl. 2016;115(10):38–43.

14. Omotosho A, Emuoyibofarhe J, Ayegba P, Meinel C. E-Prescription in Nigeria: A Survey. J Glob Pharma Technol. 2018;

15. Yang M, Shao Z, Liu Q, Liu C. Understanding the quality factors that influence the continuance intention of students toward participation in MOOCs. Educ Technol Res Dev. 2017;65(5):1195–214.

16. Crosson JC, Etz RS, Wu S, Straus SG, Eisenman D, Bell DS. Meaningful use of electronic prescribing in 5 exemplar primary care practices. Ann Fam Med. 2011;9(5):392–7.

17. Bell DS, Schueth AJ, Crosson JC, Guinan JP, Wu S, Pevnick JM, et al. Pilot testing of

electronic prescribing standards. Report) Funded by Agency Healthc Res Qual. 2007;

18. Palappallil DS, Pinheiro C. Perceptions of prescribers towards electronic prescription: A pre-implementation evaluation. J Young Pharm. 2018;10(3):313.

19. Dasgupta S, Agarwal D, Ioannidis A, Gopalakrishnan S. Determinants of information technology adoption: An extension of existing models to firms in a developing country. J Glob Inf Manag. 1999;7(3):30–40.

20. Yusif S, Hafeez-Baig A, Soar J. e-Health readiness assessment factors and measuring tools: A systematic review. Int J Med Inform. 2017;107:56–64.

21. Dixon DR, Stewart M. Exploring information technology adoption by family physicians: survey instrument valuation. In: Proceedings of the AMIA Symposium. American Medical Informatics Association; 2000. p. 185.

22. Qureshi QA, Qureshi NA, Khan IU. E-Readiness: a critical factor for successful implementation of Ehealth projects in developing countries like Pakistan. Gomal Univ J Res. 2014;30(2):77–86.

23. Shah GH, Leider JP, Castrucci BC, Williams KS, Luo H. Characteristics of local health departments associated with implementation of electronic health records and other informatics systems. Public Health Rep. 2016;131(2):272–82.

24. Ethiopia - Physicians - 1960-2018 Data _ 2021 Forecast.

25. Palappallil DS, Pinheiro C. Perceptions of Prescribers towards Electronic Prescription : A Pre-Implementation Evaluation. 2018;10(3):313–7.

26. El-Dahiyat F, Kayyali R, Bidgood P. Physicians' perception of generic and electronic prescribing: A descriptive study from Jordan. J Pharm Policy Pract [Internet]. 2014;7(1):7. Available from: https://doi.org/10.1186/2052-3211-7-7

27. Suresh V, Krishnamurthy P, Lakshmi KS, Maran K. Applying technology acceptance (TAM) model to determine the factors of acceptance in out-patient information system in private hospital sectors in Chennai city. J Pharm Sci Res. 2016 Dec 1;8:1373–7.

28. Rahimi B, Nadri H, Afshar HL, Timpka T. A systematic review of the technology

acceptance model in health informatics. Appl Clin Inform. 2018;9(3):604.

29. Khatun F, Heywood AE, Ray PK, Hanifi SMA, Bhuiya A, Liaw S-T. Determinants of readiness to adopt mHealth in a rural community of Bangladesh. Int J Med Inform. 2015;84(10):847–56.

30. Spil TAM, Schuring RW, Michel-Verkerke MB. Electronic prescription system: do the professionals use it? Int J Healthc Technol Manag. 2004;6(1):32–55.
