# Supplementary material for: Perception of physicians towards electronic prescription system and associated factors at resource limited setting 2021: Cross sectional study
Source: PLoS One. 2022 Mar 18;17(3):e0262759. doi: 10.1371/journal.pone.0262759 (PMC8932612; doi:10.1371/journal.pone.0262759)
Supplement: Attachment — Submitted filename: comments to authors review PLOS ONE.docx [file pone.0262759.s004.docx]

Comments to authors

1- This sentence in the abstract need to be revised Grammarly “ This medical error is even more serious in developing countries that lack a technologically supported healthcare system”
2- Please rewrite this sentence and organize it “ In Ethiopia because of the traditional paper-based

prescription system the overall medication prescribing error rate of 58.07%, in which incomplete

prescriptions and dosing errors were the most prevalent error types which could be prevented if there were an electronic prescription system”

3- I gave 2 examples of grammars errors I suggest to check all manuscript language to make the language more academic. There are several grammars errors the manuscript need to be checked Grammarly.
4- “ A study released in 2016 found that medical error is the third leading cause of death in the United States, after heart disease and cancer.” Please add reference after this sentence
5- “ the medical death rate” this phrase is not accurate I suggest called it medical errors related death rate
6- “ Medication errors are the leading causes of avoidable patient harm in the health care system across the world” add reference
7- “ the overall medication error in Ethiopia was found to be 57.6%” this sentence is not clear 57.6% from total prescriptions? Or from total medical errors or ….
8- “ Indeed, studies show significant improvements associated with e-prescription implementation, including an 86% decrease in serious medication errors, and an increase in Medicare formulary adherence from 14% to 88% [8].” Authors started with studies show… but only one reference was added
9- In study design and settings section, there are many unneeded details that can be shown in the questioner directly, I suggest to reduce the details about the survey and just refer the reader to the questionnaire in the supplementary files. Just summarize the domains of the survey
10- “	ethical clearance was obtained from the Debre Markos university ethical review board.” I suggest adding the number of ethical approval letter
11- In table 5, the p value of (Current computer usage) is not written
12- “ The study was done amongst the prescribing doctors at public health hospitals which has a high workload with physicians to population ratio of .0769/1000” I suggest change amongst to among. Additionally, I don’t know the meaning of numbers followed the sentence.
13- “ The finding of the current study was consistent with this studies” this sentence in discussion has grammar mistakes
