# Supplementary material for: Perception of physicians towards electronic prescription system and associated factors at resource limited setting 2021: Cross sectional study
Source: PLoS One. 2022 Mar 18;17(3):e0262759. doi: 10.1371/journal.pone.0262759 (PMC8932612; doi:10.1371/journal.pone.0262759)
Supplement: Attachment — Submitted filename: Response to Reviewers.docx [file pone.0262759.s007.docx]

Thank you for giving as the opportunity to submit a revised draft of my manuscript titled “Perception of physicians towards electronic prescription system and associated factors at resource limited setting” we appreciate the time and effort that you and the reviewers have dedicated to provide valuable feedback on the manuscript. We are grateful to the reviewers for their insightful comments on our paper. We have been able to incorporate changes to reflect most of the suggestions provided by the reviewers. We have highlighted the changes within the manuscript.

Here is a point-by-point response to the reviewers’ comments and concerns.

Comments from reviewer 1

Comment 1: It wasn’t clear how the authors would did the reliability test on such a yes/no type of questions!

Response: Thank for pointing out such comments, but most of the question in the study were Likert scale type, beside this reliability/validity test can be done for Yes or No type questions.

**Comments from Reviewer 2**

Critical comments by reviewer 2

- **Comment 1:** There is no mention of ethical committee approval and informed consent provided by subjects.
- **Response:** Thanks for the comment but we have already included this point under Ethical consideration section of the manuscript at page 8.
- **Comment 2:**  Data is not fully available (please upload to approved platforms and provide the links)
- **Response:** We will provide all the data needed
- **Comment:** Methods should describe how the investigators sent/filled the questionnaires (web-based, paper, or other)
- **Response:** As it was already stated in method section page 7 the data was collected using self-administered questionnaire(data collector provided paper printed questionnaire for participants)

**Major comments by reviewer 2**

- **Comment 1:** The questionnaire should be annexed as applied in its full version (translated from Amharic if needed)
- **Response:** we have provided the questionnaire under annex section page but we the questionnaire wasn’t translated to in Amharic we used the English questionnaire as it is.
- **Comment 2:** With the complete questionnaire annexed, the description in the "methods" section can be rewritten with a more general description.

**Response:** thanks for reminding us to revise this section we made the revision and highlighted the change.

- **Comment 3:** There are many types of e-prescription platforms. The authors need to describe what type of e-prescription is being asked about

**Response:** Thank you for raising such important question, the type e-Prescription method asked about is an electronic form sent directly to the hospital network pharmacy.

**Comments from Reviewer 3**

Comment 1: Sample size calculation is not clear

Response: Thanks for the question again, to clarify the issue we used single population proportion formula to calculate the sample size. We took 50% because there was no previous study conducted in the study area.

Sample size (n) =$\frac{({Z\frac{\alpha}{2})}^{2}x p(1-P)}{d^{2}}$, (n) =$\frac{({1.96)}^{2}x 0.5(1-0.5)}{{(0.05)}^{2}}$= 384.2

Where;

- n=estimated sample size
- p=single population proportion (50%).
- Z_α/2_ =is value of standard normal distribution (Z-statistic) at the 95% confidence level (α=0.05) which is 1.96,
- d=is the margin of error 5% (0.05)

Comment 2: The authors should first explain how they obtained the names of all participants, so as to select 384.
Response: After the sample size was determined proportional allocation was made for each hospital and the names of the physicians was collected from HRIS office simple random sampling was made

### Comment 3………authors only recruited 302 respondents (despite the computed sample size of 384). Moreover, as mentioned above, the non-response rate was over 21%andnot10%. Response: Your point here is valid and acceptable, we agree that the statement in the result section which state” A total of 302 physicians were included” is a little bit confusing.

Here we mean that a total of 302 valid response were received from participant (Total of 367 response were received but 52 of the response were incomplete and the other 13 contains some personal identification like name of the physician so we discarded 65 responses), in addition to this there were 17 questionnaires which were not returned back. But we distributed 384 questionnaires

Comment 4…there seems to be a strong bias towards young doctors, the authors should acknowledge this bias as a strong limitation

Response: This is an important issue and we accept your point to some extent and we acknowledge the bias under limitation section in the revised manuscript. But there was undeniable reason behind this, as stated in the previous manuscript the study was done in two zones namely East Gojjam and west Gojjam which has 17 public hospitals (1 referral hospital, 2 General hospitals and 14 primary hospitals). So most of the study participants were from primary hospitals, and the minimum physician professional requirement of primary hospital in Ethiopia is general practitioner. That was the case.

Comment 5: adjusted odds ratios values are reported, but there is no information on the models developed (only one? Several?)..

Response: Thank for pointing out such critical point, we have mentioned that we done binary logistic regression and multivariable logistic regression but it lacks detail so we have included the detail under data processing and analysis section line 6 to 10.

**Comments from Reviewer 4**

Comment 1: Define medical error in introduction and include the most common medical errors observed in	your region.

Response: Thank for suggesting this important point, we incorporated your suggestion in the introduction section line 5-7.

Comment 2: Even though perceived usefulness of electronic prescription was assessed, the questionnaire……

Response: Your point is valid but we have already collected the data, at this time we can’t include your suggestion. Beside this we have used perceived usefulness questionnaire from Technology acceptance model which doesn’t include previous experience questions.

**Comments from Reviewer 5**

Comment 1: According to the findings of this study, 89 percent of participants believe that paper-based prescriptions are prone to errors, however the literature mentioned [6] …………. Isn't it true that if a paper-based prescription is prone to drug errors, it shouldn't be preferred

Response: we agree that this needs clarification, the point here is before advancement of information communication technology physicians used to prescribe drug using paper printed forms which is prone to different errors. The article 6 in the manuscript shows the history drug prescription before the invention of e-Prescription. The article did not compare the paper based prescription method with e-prescription.

Comment 2: Incorporating a formula for calculating sample size would be more useful.

Response: Thank you this valuable suggestion we have incorporated sample size calculation formula in the revised manuscript in the method section line 8-16.

Comment 3: A total of 17 referral hospitals were chosen, with each receiving a proportional allotment. What was the foundation for the proportional allocation and how was it done?

Response: As you already mentioned there are 17 hospitals (1 referral hospital, 2 general hospitals and 14 primary hospitals). In order ensure the representativeness of the sample we used proportional allocation for each hospitals.

In order to show how proportional allocation done we provide an example below

One of these hospital is Debre Markos hospital which has 132 physicians, there are a total of 465 physicians and the sample size for this study is 384. So using proportional allocation

$\frac{Total number of physcian in Debre markos hospital\left( 132 \right)*Total sample needed(384)}{Total number of physcians in 17 hospitals (465)}$= 109 participants were selected from Debre Markos referral hospital.

This formula was used to allocate sample size for all other hospitals.

Comment 4: “Do you have legible handwriting?” was asked as a yes or no question to assess current prescription practice. I believe that observing written prescriptions rather than asking questions is a better way to evaluate this assertion.

Response: Yes it would be more appropriate if we observe rather than asking them, but as stated in the manuscript we used self-administered questionnaire to collect the data. That’s why we couldn’t observe their hand writing.

Comment 5: More information on the expert panel (10 doctors), such as their qualifications, departments, and so on, should be included.

Response : Thank you for this suggestion, based on your comment we have revised the document and your suggestion is included under the method section

Comment 6: What necessary correction were done in questionnaire based on the pre-test finding?
Who gave two-days training for five data collectors) on the objective of the study and data
collection procedures? Who were the data collectors? How were their levels of education, experience, and other factors matched to ensure that the data they collected was uniform?

Response: corrections like grammatical errors, order of the questions, were corrected.

The training were provided principal investigator, data collectors were Bsc health informatics professionals with two years of working experience.

Comment 7: Please specify the number and date of the ethical approval.

Response: This is incorporated in the revised manuscript.

Comment 8: It is recommended that data and material made available in the journal's repository.

Response: We will upload the data with revised document.

**Comments from Reviewer 6**

Comment 1: A study released in 2016 found that medical error is the third leading cause of death in the United States, after heart disease and cancer.”

Response: Thank you for reminding us we missed this reference, based on your comment the reference is added.

Comment 2: Medication errors are the leading causes of avoidable patient harm in the health care system across the world” add reference

Response: Thank you for reminding us we missed this reference, based on your comment the reference is added.

Comment 3: “ the overall medication error in Ethiopia was found to be 57.6%” this sentence is not clear 57.6% from total prescriptions? Or from total medical errors or ….

Response: 57.6% represents the total medical errors including drug administration and prescription.

Comment 4: “Indeed, studies show significant improvements associated with e-prescription implementation, including an 86% decrease in serious medication errors, and an increase in Medicare formulary adherence from 14% to 88% [8].” Authors started with studies show… but only one reference was added

Response: Thanks for the suggestion. It was editorial error and it changed to study.

Comment 5: In study design and settings section, there are many unneeded details that can be shown in the questioner directly, I suggest to reduce the details about the survey and just refer the reader to the questionnaire in the supplementary files. Just summarize the domains of the survey

Response: Based on your suggestion the questions were removed in summarised under Appendix section.

Comment 7: In table 5, the p value of (Current computer usage) is not written

Response: Of course it is not written. We left it black because current computer prescription was not a significant variable based on multivariable logistic regression. Its p value was >.05.

**Comments from Reviewer 7**

Comment 1: In the Introduction section, it is stated that “A study released in 2016 found that medical error is the third leading cause of death in the United States, after heart disease and cancer”. However, no citation is given.
Response: Response: Thank you for reminding us we missed this reference, based on your comment the reference is added.

**Comments from Reviewer 9**

Comment 1: what do you mean by reliability was it calculated after the pilot, you said that the expert physicians only reviewed it.

Response: Experts of physicians with different speciality reviewed the tool or the questionnaire before data collection. Beside that we have conducted pilot study outside of 17 hospitals, and reliability test were performed, it was found to be 0.78.

Comment 2: was the survey paper base? was the survey in English?

Response: Of course it was paper based and the questionnaire was in English language.

Comment 3: total sample 302, was not mentioned in the abstract

Response: Your point here is valid and acceptable, we agree that the statement in the result section which state” A total of 302 physicians were included” is a little bit confusing.

Here we mean that a total of 302 valid response were received from participant (Total of 367 response were received but 52 of the response were incomplete and the other 13 contains some personal identification like name of the physician so we discarded 65 responses), in addition to this there were 17 questionnaires which were not returned back. But we distributed 384 questionnaires. But the total sample was 384.

**Comments from reviewer 11**

Comment 1: Why does the author select the physicians with age below 35? Need to clarify it

Response: Thanks for asking as for the clarification, as we have already stated in manuscript under result section the majority of the age group were 25-34, but it doesn’t meant that all the participants are under the age of 35. There were participants with age of above 35. The majority of the age group were 25-34 because most of the study participants were from primary hospitals, and the minimum physician professional requirement of primary hospital in Ethiopia is general practitioner which is fresh graduate that tend to be younger.

Comment 2: Why the female participants were in a ratio of 25% of total participants (302).
Response: As you have said there are only 25% female participants. This the output of the data collected.

Comment 3: What were the strength and limitations of this study must incorporate in conclusion section.

Response: Thank you for the suggestion. We have incorporated your suggestion under “limitation of the study” section.

**Comments from Reviewer 12**

Comment 1: In introduction first define the medical error, and then switch to the medication error.

Response: Thank you for reminding us such an important point missed in the previous document, we incorporated the definition of Medical error under introduction section line 5 to 7.

Comment: While putting the p-value use zero before decimal and values after the decimal should be synchronous.

Response: That was an error and it corrected.

**Comments from Reviewer 13**

Comment 1: I have challenges with how the analyses were done and results were presented.

Response: Thank you for taking you time to review our paper. But your point here is too general, under method section we have stated how the analysis were performed, the software used and the model fitted. Please make sure that you raised specific that needs clarification so that we can address your comment.

Comment 2: Did not see how some means were calculated

Response: We will look forward for your specific question on which mean calculation need clarification, otherwise it is difficult to address this question.

Comment 3: The presentation of some aspect especially the methodology found a huge cap there

Response: Thank you, we have made some revision on the methodology section. But if you still have additional issue on the revision we look forward to give clarification for your comments.

**Response for general comment from reviewer 14**

…….90% respondents answered Yes to the statement "Paper prescription is prone to error" and about 70% responded that they like paper prescriptions while 79% responded that electronic prescriptions would reduce errors and save times. All three of these statements contradict each other

Response: Thank you raising such an important comment, we agree that the statement may contradict but the question are different someone may like paper prescription because it is easy and He/She has used it many years. In addition to this they may have no skill and ability to use e-Prescription. Due to these reason they like traditional paper based prescription, but doesn’t mean that this paper based prescription is not prone to error. They like it but they acknowledge its limitation.

The third question is under perceived usefulness of e-Prescription section that shouldn’t compared with the others.

The logistic regression seems inappropriate since the outcome variable is not measurable (perception). It is unclear how perception, the outcome variable was scored as 0/1/for the logistic regression on a 5-point Likert scale.

Response: Even though the original questionnaire was 5-point Likert scale we have transformed the data in to two category which 0 and 1 negative and positive respectively. Based this result logistic regression was performed.

…… stating that internet access had a positive perception. This statement doesn't mean much if the access is inconsistent. However, it is unclear since internet access and systemic factors are not well-described

Response Here we compared perception of physicians working at hospitals those have internet connection and those has no internet connection. Based on this the result revealed that physicians in organization with internet connection to develop positive perception. We haven’t considered other factors.
