# Supplementary material for: Perception of physicians towards electronic prescription system and associated factors at resource limited setting 2021: Cross sectional study
Source: PLoS One. 2022 Mar 18;17(3):e0262759. doi: 10.1371/journal.pone.0262759 (PMC8932612; doi:10.1371/journal.pone.0262759)
Supplement: Attachment — Submitted filename: Point by point response to the reviewer.docx [file pone.0262759.s008.docx]

Thank you for giving as the opportunity to submit a revised draft of my manuscript titled “Perception of physicians towards electronic prescription system and associated factors at resource limited setting” we appreciate the time and effort that you and the reviewers have dedicated to provide valuable feedback on the manuscript. We are grateful to the reviewers for their insightful comments on our paper. We have been able to incorporate changes to reflect most of the suggestions provided by the reviewers. We have highlighted the changes within the manuscript.

Here is a point-by-point response to the reviewers’ comments and concerns.

**Comments from reviewer 1**

**Comment 1**: The manuscript needs extensive English editing

Response: Thank for pointing out such a valuable comment, we have edited the manuscript accordingly.

**Comments from Reviewer 3**

**Comment 1:** They should have sent the questionnaires to 450-500 questionnaires in order to get a valid sample size of at least 384. As such, the inappropriate sample size should be acknowledged as one of the limitations of this study.

**Response:** Thanks for the comment based on your comment we have included this limitation under conclusion section of the manuscript line 10-11.

**comments by reviewer 5**

**Comment 1:** Keywords are still not in alphabetical order

**Response:** we arranged the key words in alphabetical order.

**Comment 2:** I am still not convinced with accessing current prescription practice through the question "do you have legible handwriting?

**Response:** Your point here is valid, but as we have mentioned in the method section, we used validated tools from the previous study conducted in India.

**Comment 3:** Proportional allocation of physician/hospital need to elaborate in the manuscript as well.

**Response:** Thank you for raising such important issue that we have missed; we have incorporated this in the method section of the revised document.

**Comment 4:** Ethical approval number and date need to be specified in the manuscript.

**Response:** we have included this revision under ethical consideration section.

**In addition to the above revisions all grammatical and language usage style comment raised by the reviewers were addressed accordingly.**
